# Characterizing crystalline defects in single nanoparticles from angular correlations of single-shot diffracted X-rays

**DOI:** 10.1107/S205225252000144X

**Published:** 2020-02-19

**Authors:** Akinobu Niozu, Yoshiaki Kumagai, Toshiyuki Nishiyama, Hironobu Fukuzawa, Koji Motomura, Maximilian Bucher, Kazuki Asa, Yuhiro Sato, Yuta Ito, Tsukasa Takanashi, Daehyun You, Taishi Ono, Yiwen Li, Edwin Kukk, Catalin Miron, Liviu Neagu, Carlo Callegari, Michele Di Fraia, Giorgio Rossi, Davide E. Galli, Tommaso Pincelli, Alessandro Colombo, Shigeki Owada, Kensuke Tono, Takashi Kameshima, Yasumasa Joti, Tetsuo Katayama, Tadashi Togashi, Makina Yabashi, Kazuhiro Matsuda, Kiyonobu Nagaya, Christoph Bostedt, Kiyoshi Ueda

**Affiliations:** a Department of Physics, Kyoto University, Kyoto 606-8502, Japan; b RIKEN SPring-8 Center, Sayo, Hyogo 679-5148, Japan; c Institute of Multidisciplinary Research for Advanced Materials, Tohoku University, Sendai 980-8577, Japan; d Chemical Sciences and Engineering Division, Argonne National Laboratory, 9700 S. Cass Avenue, Argonne, IL 60439, USA; e Department of Physics and Astronomy, University of Turku, 20014 Turku, Finland; f Université Paris-Saclay, CEA, CNRS, LIDYL, 91191, Gif-sur-Yvette, France; g Extreme Light Infrastructure – Nuclear Physics (ELI–NP), Horia Hulubei National Institute for Physics and Nuclear Engineering, 30 Reactorului Street, RO-077125 Magurele, Jud. Ilfov, Romania; h National Institute for Laser, Plasma and Radiation Physics, 409 Atomistilor PO Box MG-36, 077125 Magurele, Jud. Ilfov, Romania; i Elettra – Sincrotrone Trieste S.C.p.A, 34149 Basovizza, Trieste, Italy; j Department of Physics, Università degli Studi di Milano, Via G. Celoria 16, I-20133 Milano, Italy; k Fritz Haber Institute of the Max Planck Society, Faradayweg 4–6, 14195 Berlin, Germany; l Department of Physics, ETH Zürich, Stefano-Franscini-Platz 5, 8049 Zürich, Switzerland; m Japan Synchrotron Radiation Research Institute (JASRI), Sayo, Hyogo 679-5198, Japan; n Laboratory for Femtochemistry, Paul Scherrer Institute, CH-5232 Villigen PSI, Switzerland; o LUXS Laboratory for Ultrafast X-ray Sciences, École Polytechnique Fédérale de Lausanne (EPFL), CH-1015 Lausanne, Switzerland

**Keywords:** X-ray diffraction, X-ray scattering, structure determination, single nanoparticles, crystalline defects, XFELs, angular correlations, stacking faults

## Abstract

Characterizing and controlling the uniformity of nanoparticles is crucial for their application in science and technology because crystalline defects in the nanoparticles strongly affect their unique properties. Recently, ultra-short and ultra-bright X-ray pulses provided by X-ray free-electron lasers (XFELs) opened up the possibility of structure determination of nanometre-scale matter with Å spatial resolution. However, it is often difficult to reconstruct the 3D structural information from single-shot X-ray diffraction patterns owing to the random orientation of the particles. This report proposes an analysis approach for characterizing defects in nanoparticles using wide-angle X-ray scattering (WAXS) data from free-flying single nanoparticles. The analysis method is based on the concept of correlated X-ray scattering, in which correlations of scattered X-ray are used to recover detailed structural information. WAXS experiments of xenon nanoparticles, or clusters, were conducted at an XFEL facility in Japan by using the SPring-8 Ångstrom compact free-electron laser (SACLA). Bragg spots in the recorded single-shot X-ray diffraction patterns showed clear angular correlations, which offered significant structural information on the nanoparticles. The experimental angular correlations were reproduced by numerical simulation in which kinematical theory of diffraction was combined with geometric calculations. We also explain the diffuse scattering intensity as being due to the stacking faults in the xenon clusters.

## Abstract 2

A novel analysis method is proposed for X-ray diffraction patterns recorded in single-shot wide-angle X-ray scattering experiments of free-flying single nanoparticles. The crystalline defects in the nanoparticles are characterized by analyzing the angular correlations between Bragg spots in the single diffraction patterns.

## 1. Introduction   

Nanoparticles have been employed in a wide range of fields owing to their unique optical, electronic, magnetic, chemical and catalytic properties. These properties sensitively depend on various structural parameters such as size, shape and atomic structure (Brus, 1984 ▸; Halperin, 1986 ▸; Bawendi *et al.*, 1990 ▸; Alivisatos, 1996 ▸; Burda *et al.*, 2005 ▸). Therefore, characterizing and controlling the structural uniformity of the nanoparticles is an important issue for their application in science and technology. Several techniques have been used to characterize nanoparticles. Electron microscopy provides the structural information of single nanoparticles at atomic resolution; however, it cannot probe the internal structure of large nanoparticles (with thicknesses >50 nm) and often requires highly demanding procedures for sample preparation. Conventional powder X-ray diffraction is also widely used to characterize nanoparticles; however, the structural information is inevitably averaged by the random ensemble of the particles.

Bright femtosecond X-ray pulses from X-ray free-electron lasers (XFELs) (Emma *et al.*, 2010 ▸ Ishikawa *et al.*, 2012 ▸; Ko *et al.*, 2017 ▸; Tschentscher *et al.*, 2017 ▸; Milne *et al.*, 2017 ▸) have provided novel opportunities to investigate the structure of nanoscale samples with Å resolution (Chapman *et al.*, 2011 ▸; Aquila *et al.*, 2015 ▸). In single-shot X-ray diffraction with XFELs, ultra-short X-ray pulses can avoid the major radiation-damage processes in the sample. Coherent-diffraction imaging with XFELs has enabled the structure determination of a wide variety of samples, including aerosol particles (Loh *et al.*, 2012 ▸; Starodub *et al.*, 2012 ▸), rare-gas clusters (Bostedt *et al.*, 2010 ▸; Gorkhover *et al.*, 2012 ▸), nanoparticles (Clark *et al.*, 2013 ▸; Takahashi *et al.*, 2013 ▸; Xu *et al.*, 2014 ▸; Barke *et al.*, 2015 ▸), superfluid quantum systems (Gomez *et al.*, 2014 ▸) and biological objects (Chapman *et al.*, 2011 ▸; Seibert *et al.*, 2011 ▸; Boutet *et al.*, 2012 ▸; Redecke *et al.*, 2013 ▸; Kupitz *et al.*, 2014 ▸; Hantke *et al.*, 2014 ▸; Rose *et al.*, 2018 ▸).

The analysis of single-shot diffraction data from single particles is inherently difficult owing to the random orientations of the particles in the scattering geometry. The diffraction pattern recorded on each shot is composed of scattering sensitive to only those 3D spatial frequencies which, in the representation of reciprocal space, lie on a certain 2D manifold corresponding to the Ewald sphere. Usually, the particle orientations must be determined to recover the entire 3D structural information from the diffraction patterns (Ekeberg *et al.*, 2015 ▸). In serial femtosecond crystallography (SFX) of protein crystals, the crystal orientation is successfully determined for each diffraction pattern by indexing several Bragg spots that appear in that pattern. However, the procedure used in SFX is usually not applicable to the wide-angle X-ray scattering (WAXS) data of nanoparticles with short lattice parameters (a few Å’s) because there are usually not enough Bragg spots in a pattern to obtain a reliable indexing solution.

An alternative approach to overcome the random particle orientations is a method called correlated X-ray scattering (CXS) (Kam, 1977 ▸, 1980 ▸) [also referred to as fluctuation X-ray scattering (Kam *et al.*, 1981 ▸) or angular X-ray cross-correlation analysis (Kurta *et al.*, 2017 ▸; Zaluzhnyy *et al.*, 2019 ▸)]. CXS is an emerging method employed to recover the structure of an object from X-ray diffraction patterns of a random ensemble of identical objects. In CXS measurements with XFELs, the particles are frozen in space throughout the snapshot exposures of femtosecond X-ray pulses. The resulting diffraction patterns are anisotropic and contain intensity variations, which provide further structural information beyond diffraction patterns recorded with conventional sources, with exposure times that span many configurations of the fluctuating sample and which average away the short-exposure variations. Recently, several CXS studies at XFELs (Chen *et al.*, 2012 ▸; Liu *et al.*, 2013 ▸; Malmerberg *et al.*, 2015 ▸; Kurta *et al.*, 2017 ▸) have been carried out, which have demonstrated the structure reconstruction of nanoscale samples in solution or on a substrate. Mendez *et al.* applied CXS to a WAXS experiment with XFELs (Mendez *et al.*, 2014 ▸, 2016 ▸) and demonstrated the effectiveness of CXS in the wide-angle region. They developed a robust analysis technique that could effectively extract intensity correlations from single-shot diffraction patterns and applied the method to the WAXS data of tens of thousands of gold nanoparticles in a solution. The analysis revealed evident angular correlation in the Debye–Scherrer rings, which offered information on crystal twinning in the particles.

In this report, we propose an application of CXS for characterizing crystalline defects in free-flying nanoparticles. The present method analyzes single-particle diffraction patterns with more than one Bragg spot and extracts the angular correlations between pairs of Bragg spots in each image. The method was applied to the analysis of WAXS data of single xenon (Xe) nanoparticles recorded by single-shot X-ray diffraction using the Spring-8 Angstrom Compact free-electron LAser (SACLA)  (Ishikawa *et al.*, 2012 ▸) in Japan. The extracted angular correlations contain rich information on the crystalline structure of the nanoparticles, which cannot be accessed by conventional orientationally averaged diffraction data. The angular correlations were well reproduced by numerical simulation based on geometric calculations, following which we successfully characterized the stacking faults in the nanoparticles (Ferguson, 2016 ▸).

## 2. Method   

### 2.1. XFEL   

The experiments were performed at experimental hutch 2 of beamline 3 (Yabashi *et al.*, 2017 ▸) of the SACLA. For details of the experimental setup, see also our previous publication (Nishiyama *et al.*, 2019 ▸). The SACLA generated 1.1 Å X-ray pulses at a repetition rate of 30 Hz. The pulse duration was estimated to be 10 fs [full width at half-maximum (FWHM)]  (Inubushi *et al.*, 2012 ▸). The XFEL pulses were focused on the reaction point by a set of Be compound reflection lenses (CRLs) (Katayama *et al.*, 2019 ▸). The focused XFEL beam size was measured to be ∼1.5 µm (FWHM). The X-ray fluence was evaluated to be 2 × 10^10^ photons µm^−2^ by considering the transmittance of the beamline and the Be CRLs.

### 2.2. Xe clusters   

Xe clusters were prepared by adiabatic expansion of Xe gas through a nozzle (Bostedt *et al.*, 2012 ▸) with 200 µm diameter and 4° half-opening angle. The nozzle temperature was 290 K, the stagnation pressure was 3 MPa and the average cluster size was estimated to be 〈*N*〉 ≃ 1.6 × 10^7^ atoms (radius 〈*r*〉 ≃ 60 nm) according to a known scaling law (Hagena, 1981 ▸). On average, the number of clusters contributing to each X-ray shot was less than one. In these experimental conditions, the sample intercepted an area ∼0.6% of that of the beam spot and therefore it can be assumed to be uniformly illuminated by coherent radiation with about 10^8^ photons.

### 2.3. Collecting X-ray diffraction patterns   

The scattered X-rays from the Xe clusters were recorded on a shot-by-shot basis with a multi-port CCD sensor detector (Kameshima *et al.*, 2014 ▸) located 100 mm downstream of the reaction point. We collected 573 089 images in total. An averaged dark image was subtracted from the images. Images with Bragg spots were identified and selected using a blob-finding algorithm (Bradski, 2000 ▸). The statistics of the events are described in Table 1 ▸. The number of images containing just one Bragg spot and more than one Bragg spot were 45 843 (∼8%) and 3 984 (∼0.7%), respectively.

### 2.4. Angular correlation of two Bragg spots   

**Figure 1:**
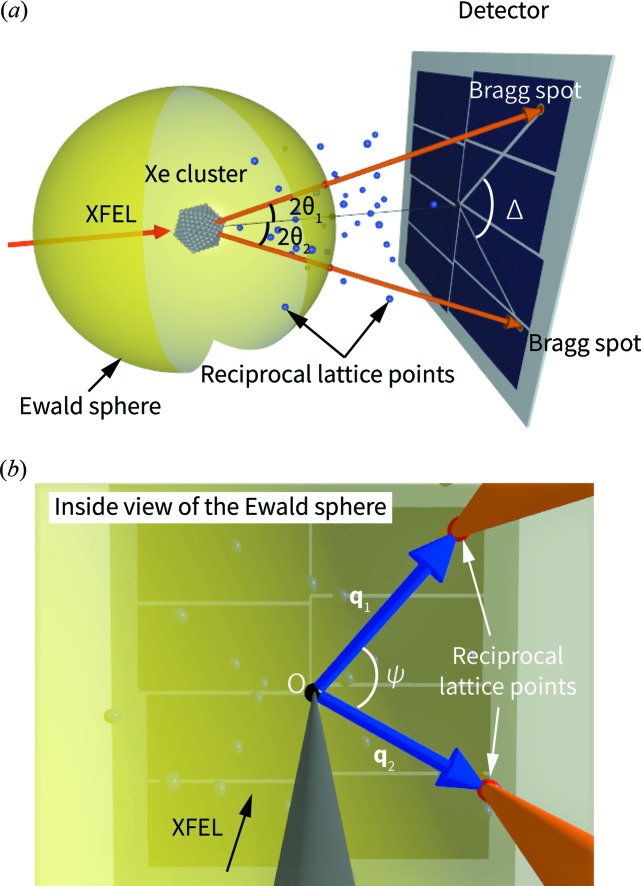
The geometry in reciprocal space together with the experimental configuration. (*a*) Ewald sphere (yellow) and reciprocal lattice points (navy blue and red) are depicted with the experimental configuration. θ_1_ and θ_2_ are Bragg angles and Δ is the azimuthal angular separation between the two Bragg spots. (*b*) Inside view of the Ewald sphere. ψ is defined as the angle between the two corresponding momentum-transfer vectors: **q**_1_ and **q**_2_.

Fig. 1 ▸(*a*) depicts a schematic of the Ewald sphere (yellow) and the reciprocal lattice points (navy blue and red). Here we assume diffraction of a large-size perfect crystal. The reciprocal lattice points rotate about the origin of the reciprocal space depending on the crystal orientation. When a reciprocal lattice point lies on the surface of the Ewald sphere [red points in Figs. 1 ▸(*a*) and 1(*b*)] it gives rise to a Bragg spot on the detector. Furthermore, when another reciprocal lattice point also lies on the surface of the Ewald sphere another Bragg spot will be observed in the diffraction image. The probability of observing two Bragg peaks from a single crystal is lower than that of observing a single Bragg spot. When two Bragg spots from a single crystal do occur, their positions on the detector are correlated, as they are determined by the two corresponding momentum-transfer vectors [blue arrows in Fig. 1 ▸(*b*)].

Here, we describe the procedure of the angular-correlation analysis. First, we selected a certain arbitrary combination (with repetition allowed) of a pair of Debye–Scherrer rings in the powder diffraction pattern that can be formed by summing many single-shot diffraction images. We filtered diffraction images with Bragg spots located on the selected rings and used them for the angular-correlation analysis. In each diffraction image, the azimuthal angular separation Δ between two Bragg spots was calculated [see Fig. 1 ▸(*a*)]. We define ψ as the angle between the two corresponding momentum-transfer vectors, **q**_1_ and **q**_2_, as follows, 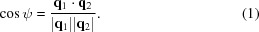
Cos ψ is calculated using the following relation (Mendez *et al.*, 2014 ▸, 2016 ▸), 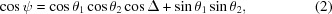
where θ_1_ and θ_2_ are the Bragg angles at wavelength λ, as follows, 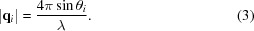
Note that ψ approaches Δ in the small-angle limit θ_1_, θ_2_ → 0. The same procedure was iterated for pairs of Bragg spots in all diffraction images that were identified to contain Bragg spots and the correlation function *C* (cos ψ) was calculated as 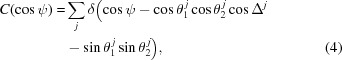
where *j* is an index for pairs of the Bragg spots and δ is the Dirac delta function. When an image contained more than two Bragg spots, cos ψ was evaluated for all combinations of the Bragg spots.

## 3. Results and discussions   

### 3.1. X-ray diffraction pattern   

**Figure 2:**
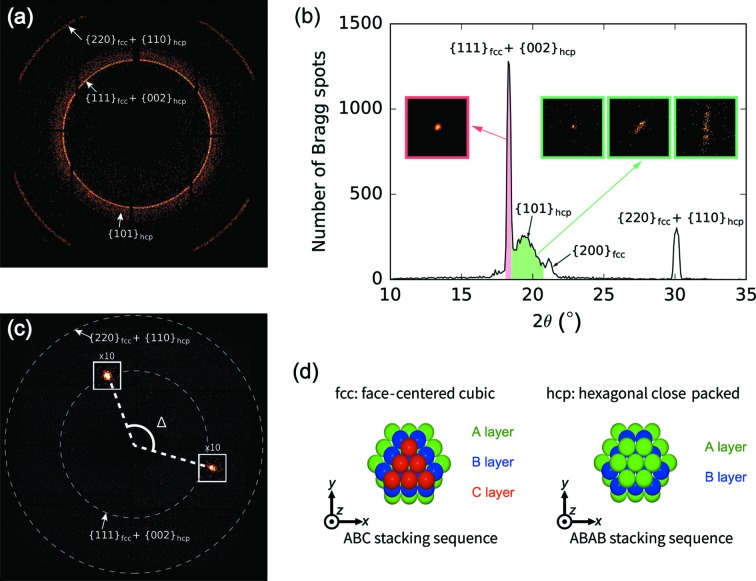
Distribution of the Bragg spot positions on the detector (*a*) and as a function of 2θ (*b*). Three sharp peaks are observed at 2θ ≃ 18.3, 21.1 and 30.1°. These peak positions correspond to {111}_fcc_ + {002}_hcp_, {200}_fcc_ and {220}_fcc_ + {110}_hcp_, respectively. In addition, one broad peak is observed at 2θ ≃ 19.4°, which corresponds to the {101}_hcp_ position. Note that the yield of the {220}_fcc_ + {110}_hcp_ peak is undervalued because of the limit on the detection region. The angular correlations were calculated using spots lying on the {111}_fcc_ + {002}_hcp_ peak (18.1° < 2θ < 18.5°, filled with pink) and the {101}_hcp_ peak (18.5° < 2θ < 20.7°, filled with green). Insets show the images of the Bragg spots located on the {111}_fcc_ + {002}_hcp_ and {101}_hcp_ peaks. (*c*) A representative single-shot image used for the angular-correlation analysis. The image contains two Bragg spots on the {111}_fcc_ + {002}_hcp_ and {101}_hcp_ rings. The areas marked by white rectangles are zoomed in (×10). (*d*) F.c.c. and h.c.p. stacking sequences.

Fig. 2 ▸(*b*) shows the distribution of the Bragg spot positions in the collected images as a function of the scattering angle 2θ. This angle-integrated powder pattern displays three sharp peaks at positions corresponding to the {111}_fcc_ (face-centered cubic) + {002}_hcp_ (hexagonal close packed) reflections, the {200}_fcc_ reflections and the {220}_fcc_ + {110}_hcp_ reflections. In addition to those sharp peaks, a broad peak at 2θ ≃ 19.4° is also observed. The position of this broad peak corresponds to the {101}_hcp_ reflection. It should be emphasized that the broad peak originates from bright spots distributed over a range of 2θ angles on the detector. This fact indicates that the broad peak originates from some long-range structural order in the Xe clusters. The coexistence of the f.c.c. and h.c.p. diffraction peaks has already been reported in previous diffraction experiments on rare-gas clusters (Waal *et al.*, 2000 ▸; Danylchenko *et al.*, 2004 ▸; Ferguson, 2016 ▸; Ferguson *et al.*, 2016 ▸). These two structures only differ by the stacking sequence [*ABC* for f.c.c. and *ABAB* for h.c.p., see Fig. 2 ▸(*d*)], and the free-energy difference is very small. Therefore, a mixture of these structures along the stacking direction is commonly observed in various materials (Kittel, 2004 ▸).

The insets in Fig. 2 ▸(*b*) show some representative images of the Bragg spots located on each of the corresponding Debye–Scherrer rings. Each Bragg spot typically consisted of <200 photons. Most of the Bragg spots located on the {111}_fcc_ + {002}_hcp_ ring are circular shaped. On the other hand, elliptical and streaked spots are sometimes observed in the broad {101}_hcp_ ring. These anisotropic intensities originate from diffuse intensity distribution in reciprocal space, implying crystalline defects in the clusters. However, reconstructing the 3D structure factor from the single-shot diffraction patterns is not straightforward owing to the random orientations of the particles. In this article, we employ an angular-correlation analysis to extract meaningful information from the diffraction patterns of randomly oriented particles.

### 3.2. Angular correlations of two Bragg spots   

By applying an angular-correlation analysis to shot-by-shot diffraction images, we can obtain further structural details beyond what is possible with a 1D powder diffraction pattern. A representative image used for the angular-correlation analysis is shown in Fig. 2 ▸(*c*). Our angular-correlation analysis was applied in the cases of Bragg spots lying on the rings corresponding to the {111}_fcc_ + {002}_hcp_ reflections (18.1° < 2θ < 18.5°) and the {101}_hcp_ reflections (18.5° < 2θ < 20.7°) [see Fig. 2 ▸(*a*)]. For convenience, we refer to these rings (and their corresponding peaks in the 1D plot) as {111}_fcc_ and {101}_hcp_, respectively. Note that the {220}_fcc_ + {110}_hcp_ ring was not used for the angular-correlation analysis because we could not record the whole ring owing to the limited detection area. Angular correlations were calculated for three combinations of pairs of rings: {101}_hcp_ − {101}_hcp_, {111}_fcc_ − {111}_fcc_ and {101}_hcp_ − {111}_fcc_. We adopted a lower threshold for the blob-finding algorithm than that used in the calculation of the 1D powder diffraction pattern because the angular-correlation analysis is less susceptible to false spots owing to noise in the images. In addition, we excluded eight images containing more than five Bragg spots in any of the selected rings, which otherwise result in uncorrelated noise in the angular correlations.

**Figure 3:**
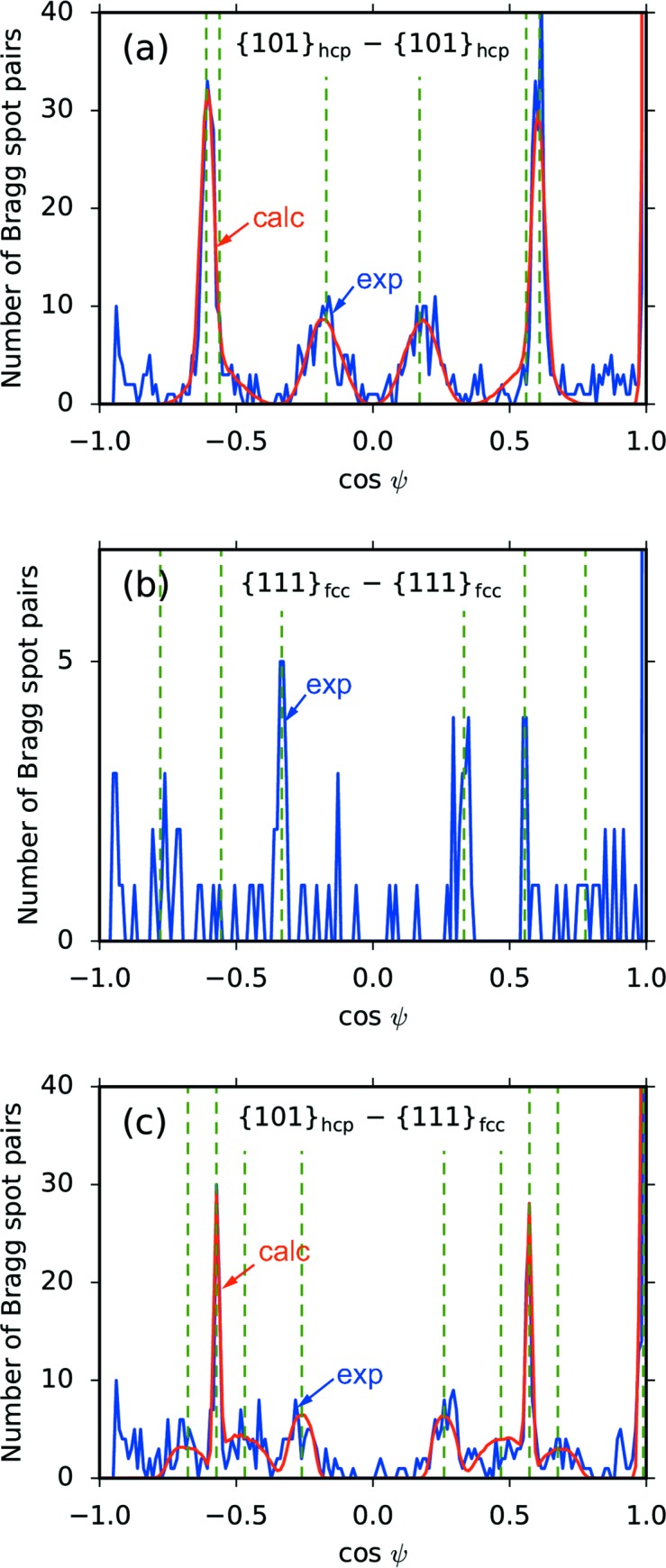
Results of angular-correlation analysis. The number of Bragg spot pairs are shown as functions of cos ψ. Blue lines depict the experimental angular correlations of (*a*) {101}_hcp_ − {101}_hcp_, (*b*) {111}_fcc_ − {111}_fcc_ and (*c*) {101}_hcp_ − {111}_fcc_. Green dashed lines depict the peak positions of the angular correlations calculated by evaluating cos ψ for the two corresponding reciprocal lattice vectors (see Appendix *A* [App appa]). Numerical simulations were performed to account for the peak broadening in (*a*) and (*c*) (red lines). The details of the simulation are provided in the main text. The optimized parameters in the simulation are σ_*xy*_ = 0.02 Å^−1^ and σ_*z*_ = 0.25 Å^−1^.

The angular correlation of {101}_hcp_ − {101}_hcp_ is indicated by the blue line in Fig. 3 ▸(*a*). Note that the correlation around cos ψ ≃ −1 is not available because of the geometrical limit of ψ  (Mendez *et al.*, 2016 ▸). The peak positions in the angular correlation agree with the prediction [green dashed lines in Fig. 3 ▸(*a*)] made by evaluating cos ψ for two {101}_hcp_ reciprocal lattice vectors (see Appendix *A*1[App appa] [Sec seca1]). In addition, the peaks in the angular correlation are broad. If one assumes perfect crystals with infinite volume, the structure factor has sharp peaks at the reciprocal lattice points; consequently, the angular correlation would also have sharp peaks. Therefore, the peak widths in Fig. 3 ▸(*a*) indicate a structure factor with a diffuse intensity distribution around the reciprocal lattice points.

Fig. 3 ▸(*b*) shows the angular correlation for {111}_fcc_ − {111}_fcc_. In contrast to the frequent occurrence of pairs of {101}_hcp_ Bragg spots, the number of pairs of {111}_fcc_ Bragg spots is much less. This is surprising if one considers the huge number of recorded {111}_fcc_ spots [see Fig. 2 ▸(*b*)]. One might consider that this is because of the geometrical tolerance of the Bragg conditions. If the structure factor has sharp peaks around the reciprocal lattice points, the crystals must be oriented very accurately in order to give rise to two correlated Bragg spots. We will return to this point later in Section 3.4. The correlation has small but significant peaks at cos ψ = −1/3, 1/3 and 5/9. The significance of these peaks was 5.8σ, 4.7σ and 4.3σ, respectively, which were calculated by assuming a noise level of 0.5 pairs bin^−1^. These peak positions agree with the prediction considering reciprocal lattice points for f.c.c. crystals with twinned faults (Mendez *et al.*, 2016 ▸) (see also Appendix *A*2[App appa] [Sec seca2]). Furthermore, the number of uncorrelated Bragg spot pairs should also be noticed. Uncorrelated Bragg spot pairs can originate from polycrystalline structures with randomly oriented crystalline domains or multiple clusters in the XFEL focus. In the present case, the number of uncorrelated Bragg spot pairs is reasonably explained by the presence of multiple clusters in the XFEL focus. This fact implies that most of the Xe clusters do not form polycrystalline structures.

The {101}_hcp_ and {111}_fcc_ Bragg spots show clear angular correlation [see Fig. 3 ▸(*c*)]. The clear correlation indicates that pairs of {101}_hcp_ and {111}_fcc_ Bragg spots originated from single particles and not from independent particles with different crystalline structures. The vertical green dashed lines in Fig. 3 ▸(*c*) indicate the peak positions predicted by considering the f.c.c.–h.c.p. mixture structure along the stacking direction (see Appendix *A*3[App appa] [Sec seca3]). The peak positions are successfully explained by the prediction. Furthermore, peaks in the angular correlation exhibit specific peak widths. As will be seen later, the non-uniform widths of the angular-correlation peaks result from an anisotropic diffuse intensity around the reciprocal lattice points.

### 3.3. Numerical simulation of angular correlations   

We developed a numerical simulation method to model and fit the intensity distribution around the reciprocal lattice points. In the simulation, we considered 3D peak broadening around the {101}_hcp_ reciprocal lattice points, which are expressed as a sum of Gaussian functions, 
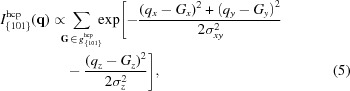
with the *z* axis along the [111]_fcc_ direction. 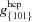 is the set of {101}_hcp_ reciprocal lattice vectors. The angular correlation was modeled with the following function,
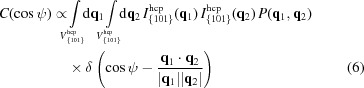
and
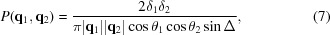
where *P*(**q**_1_, **q**_2_) is the probability that two small spheres around **q**_1_ and **q**_2_ with radii of δ_1_ and δ_2_, respectively, intersect with the Ewald sphere simultaneously. The integral in equation (6) was taken over the region 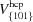, which specifies the momentum-transfer region of {101}_hcp_ used to construct the angular correlation. We used the Monte Carlo method to calculate the integral, in which reciprocal lattice vectors were perturbed randomly with a Gaussian distribution, as represented in equation (5). The width parameters, σ_*xy*_ and σ_*z*_, as well as the constant of proportionality in equation (6) were optimized to reproduce the experimental angular correlation in Fig. 3 ▸(*a*).

### 3.4. Stacking faults in the Xe clusters   

**Figure 4:**
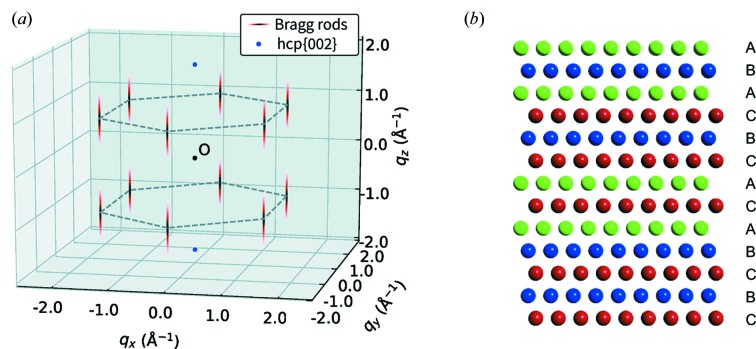
(*a*) Structure factor when α = 0.5. The structure factor has 12 Bragg scattering rods (red rods) around the {101}_hcp_ reciprocal lattice points and two sharp peaks (blue points) at the {002}_hcp_ reciprocal lattice points. The integral breadth β of the Bragg scattering rods is theoretically calculated to be 0.59 Å^−1^. (*b*) An example of the r.h.c.p. structure in the Xe clusters.

The red line in Fig. 3 ▸(*a*) depicts the simulation results with the optimized parameters: σ_*xy*_  =  0.02 Å^−1^ and σ_*z*_   =  0.25 Å^−1^. The simulation results successfully reproduced the experimental angular correlation, including the characteristic widths of the peaks. The optimized parameters suggest that the structure factor has a rod-like intensity distribution around the {101}_hcp_ reciprocal lattice points which extends in the [111]_fcc_ direction, parallel to the [001]_hcp_ direction [see Fig. 4 ▸(*a*)]. The rod-like intensity distributions are called Bragg scattering rods. We also performed a similar simulation for the {101}_hcp_ − {111}_fcc_ correlation [red line in Fig. 3 ▸(*c*)] and we successfully explained the experimental result by assuming the same intensity distributions around the {101}_hcp_ reciprocal lattice points described with the same parameters σ_*xy*_ and σ_*z*_, as in the previous case (see Appendix *B* [App appb] for details).

Our findings from the angular-correlation analysis are supported by the profiles of the Bragg spots in the single-shot diffraction patterns. In fact, the elliptical and streaked spots observed in the {101}_hcp_ peak region are parts of the Bragg scattering rods resulting from the stacking faults. The profiles of the Bragg spots vary depending on the orientation of the crystal and hence on the orientation of the reciprocal lattice in reciprocal space, *i.e.* how the Bragg scattering rods intersect the Ewald sphere. Streaked patterns are observed when one of the Bragg scattering rods is nearly in contact with and tangential to the Ewald sphere. The single-shot diffraction patterns encode structural information on single particles and it is possible to discuss the particle-by-particle structural information using the single-shot diffraction patterns. However, it is challenging to retrieve the structural information from the single-shot diffraction data and such analysis is beyond the scope of this study.

To understand the origin of the diffuse scattering intensity, we employ the diffraction theory of close-packed crystals containing stacking faults (Paterson, 1952 ▸). According to the theory, when a crystal contains stacking faults, two types of peak broadening occur in reciprocal space. Here, we use the notation (*hkl*)_hcp_ to represent the reflections. For reflections that satisfy the condition *h* − *k* = 3*n* (*n* = an integer), the peak widths are not affected by the stacking order and the widths reflect the size of the entire close-packed crystal (through an inverse relationship). On the other hand, if *h* − *k* = 3*n* ± 1, the widths depend on the stacking order. The structure factor has a broad intensity distribution along the [001]_hcp_ direction, which results in the emergence of Bragg scattering rods. Theoretically, the degree of stacking faults is described with a parameter α, which is the probability that the *N*th and (*N* + 2)th layers have different stacking positions *ABC*. α = 1 corresponds to f.c.c., α = 0 to h.c.p. and α = 0.5 to random hexagonal close packed (r.h.c.p.) structure.

The experimental results agree well with the r.h.c.p. structure: α = 0.5. Fig. 4 ▸(*a*) shows the schematics of the 3D structure factor when α = 0.5. The figure depicts only the intensities that are related to the discussion of the angular correlations. The structure factor has two sharp peaks at {002}_hcp_ [*i.e.* (111)_fcc_ and 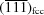] reciprocal lattice points [blue points in Fig. 4 ▸(*a*)]. In addition, there are 12 Bragg scattering rods that have broad intensity distributions around the {101}_hcp_ reciprocal lattice points [red rods in Fig. 4 ▸(*a*)]. Here, we verify the consistency between the experimental results and the structure factor when α = 0.5. First, the structure factor is in agreement with the 1D diffraction pattern. The sharp ring at 2θ ≃ 18.3° originates from the sharp intensity at {002}_hcp_ and the broad ring at 2θ ≃ 19.4° originates from the Bragg scattering rods. The slight {200}_fcc_ ring at 2θ ≃ 21.1° cannot be explained by the structure factor and the observation of this ring suggests the existence of a small amount of nearly perfect f.c.c. crystals. However, it has little influence on the following discussion on the angular correlations. Secondly, peak positions in the angular correlations agree with the structure factor. As mentioned above, the peak positions are successfully explained by considering {101}_hcp_ and {111}_fcc_ reciprocal lattice points, and the structure factor has finite intensity around the reciprocal lattice points. Thirdly, the diffuse intensities around the {101}_hcp_ reciprocal lattice points are also consistent with the random stacking case. According to the theory (Paterson, 1952 ▸), the integral breadth of the Bragg scattering rods, which is defined as the ratio of the integrated intensity along a rod to the maximum intensity, is expressed as follows, 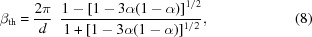
where *d* = *a* ^fcc^(3)^−1/2^ is the spacing between two close-packed layers and *a* ^fcc^ = 6.14 Å is the lattice constant of the f.c.c. crystal. Substituting α = 0.5 into equation (8), the integral breadth of the Bragg scattering rods is calculated to be β_th_ = 0.59 Å^−1^. This value is in reasonable agreement with the value estimated from the angular correlations β_exp_ = (2π)^1/2^σ_*z*_ = 0.63 Å^−1^. It is noteworthy that the peak broadening caused by the finite crystal size is almost negligible compared with that caused by the stacking faults. Finally, we can explain the small amount of correlated Bragg spot pairs in the {111}_fcc_ − {111}_fcc_ correlation. Most of the {111}_fcc_ Bragg spots originate from the sharp peaks at {002}_hcp_ and no correlated Bragg spot pairs are detectable from these two peaks. The slight correlation is because of the contamination from the Bragg scattering rods; we cannot distinguish the {002}_hcp_ Bragg spots from those originating from the Bragg scattering rods.

The present method provides new insight into the structure of nanoparticles. The angular-correlation analysis revealed that in the present case of large Xe clusters they do not form a multi-domain structure but form single close-packed crystals with random stacking orders. Previous studies suggested that small rare-gas clusters form multiply twinned structures with fivefold symmetry (Farges *et al.*, 1983 ▸). However, in the present study, we found no evidence of fivefold twinning. This might imply a structural transition in the growth process from the fivefold twinned structure to the non-twinned structure. The possibility of an r.h.c.p. structure in rare-gas clusters has already been suggested in a previous study on Ar clusters (with 〈*N*〉 ≃ 80 000 atoms) (Waal *et al.*, 2000 ▸). The present study suggests that even much larger clusters (〈*N*〉 ≃ 10^7^ atoms) can form an r.h.c.p. structure. The formation of an r.h.c.p. structure appears to be related to the growth kinetics of the rare-gas clusters.

## 4. Summary and outlook   

In summary, we explored the structural properties of Xe clusters by means of single-shot WAXS data from free-flying single nanoparticles as recorded at an XFEL. Our data revealed evident angular correlations between the Bragg spots in the single-shot diffraction patterns. We compared the observed angular correlations with the results of a newly developed simulation code, which combines the theory of diffuse X-ray scattering with geometrical considerations. From the comparison, we found evidence for an r.h.c.p. structure in the Xe nanoparticles. The results of this study on Xe nanoparticles validate our novel approach for structural analysis and characterization of defects in individual 3D nanoparticles based on single-shot X-ray diffraction data of free-flying atomic clusters and simulations.

## Figures and Tables

**Table 1:** Statistics of the events The numbers of X-ray shots with hits are listed together with those used for the angular-correlation analysis. Note that the event rate is overestimated because of the low threshold in Bragg spots detection suitable for the angular-correlation analysis.

	Number of X-ray shots	Event rate (%)
Total X-ray shots	573089	
Images with hit(s)	45843	8
Images with >1 hit(s)	3984	0.7
101_hcp_ − 101_hcp_	802	0.1
111_fcc_ − 111_fcc_	148	0.03
101_hcp_ − 111_fcc_	456	0.08

## Appendix A Peak positions in angular correlations

### A1. {101}_hcp_ − {101}_hcp_

The peak positions in the angular correlation are calculated as follows. First, we define the primitive reciprocal lattice vectors for h.c.p.,

where *a* ^hcp^ and *c* ^hcp^ = (8/3)^1/2^ *a* ^hcp^ are lattice constants of the h.c.p. crystal. Reciprocal lattice vectors  are expressed by the linear combinations of the primitive reciprocal lattice vectors,

where *h*, *k* and *l* are integers. The {101}_hcp_ reflection has 12 equivalent reciprocal lattice vectors:

The peak positions are obtained by calculating cos ψ for all combinations of two vectors from ,

### A2. {111}_fcc_ − {111}_fcc_

The peak positions in the {111}_fcc_ − {111}_fcc_ correlation are calculated in a similar manner to the {101}_hcp_ − {101}_hcp_ correlation. The primitive reciprocal lattice vectors of f.c.c. and the reciprocal lattice vectors are

and

The set of {111}_fcc_ reciprocal lattice vectors is defined as .

When the f.c.c. crystal contains a twinned fault (*i.e.* expressed by a stacking sequence: *…ABCABCBACBA…*), we should consider an extended set of momentum-transfer vectors, which is expressed with a reflection operator **T** across the (111)_fcc_ plane (Mendez *et al.*, 2016 ▸):

and

The peak positions are obtained by calculating cos ψ for all combinations of two vectors from .

### A3. {101}_hcp_ − {111}_fcc_

In order to predict the peak positions in the {101}_hcp_ − {111}_fcc_ correlation, we need to consider both {101}_hcp_ and {111}_fcc_ reciprocal lattice points. We assume a mixture of the f.c.c. and h.c.p. structures along the stacking direction, which is, for example, expressed by a stacking sequence: *…ABCABCABAB…*. The reciprocal lattice vectors for f.c.c., , are expressed as follows:

and

**R** is a rotation matrix for converting the coordinates to those common to h.c.p. The peak positions are obtained by calculating cos ψ for all combinations of two vectors from  and .

Note that the peak positions remain the same even when the f.c.c. crystal contains a twin fault.

## Appendix B Simulation of angular correlation

### b1. {101}_hcp_ − {111}_fcc_

The scattering intensity  around the {111}_fcc_ reciprocal lattice points is formulated as follows. We neglect the peak broadening around the {111}_fcc_ reciprocal lattice points. This is verified by the fact that the peak broadening in the angular correlations is dominated by the diffuse intensity distribution of the Bragg rods. When a crystal contains stacking faults, elements of the set  are no longer equivalent. Therefore, we should consider the scattering intensity

and

where *c*_1_ and *c*_2_ are constants. In the present case, we used *c*_1_ = 1 and *c*_2_ = 0.5 to reproduce the experimental result.

Using the expressions for  and , the angular correlation for {101}_hcp_ − {111}_fcc_ was modeled with the following function,
